# Exposure to secondhand smoke is associated with increased left ventricular mass

**DOI:** 10.18332/tid/136415

**Published:** 2021-06-04

**Authors:** Travis M. Skipina, Bharathi Upadhya, Elsayed Z. Soliman

**Affiliations:** 1 Department of Internal Medicine, Wake Forest School of Medicine, Winston Salem, United States; 2 Section of Cardiovascular Medicine, Department of Internal Medicine, Wake Forest School of Medicine, Winston Salem, United States; 3 Institute of Global Health and Human Ecology, School of Sciences and Engineering, The American University in Cairo, New Cairo, Egypt

**Keywords:** left ventricular mass, secondhand smoke, passive smoking, hypertension, end-organ damage

## Abstract

**INTRODUCTION:**

Chronic hypertension is associated with left ventricular hypertrophy. Recent evidence suggests that secondhand smoke (SHS) exposure is associated with chronic hypertension, so we sought to examine the relationship between secondhand smoke exposure and electrocardiographic left ventricular (LV) mass among non-smokers.

**METHODS:**

This analysis included 4982 non-smoker participants from the Third National Health and Nutrition Examination (NHANES-III). Non-smoking was defined by self-report and serum cotinine ≤10 ng/mL, a biomarker for tobacco exposure. SHS exposure was defined as serum cotinine level ≥1 ng/mL. LV mass was estimated using an electrocardiographic model developed and applied in NHANES-III then validated in the Cardiovascular Health Study. Multivariable linear regression was used to examine the cross-sectional association between SHS exposure (vs no exposure) with estimated LV mass index. In similar models, we also examined the associations of LV mass index across quartiles of serum cotinine (reference group, 1st quartile) and in subgroups stratified by age, race, sex, hypertension, and obesity.

**RESULTS:**

About 9.8% (n=489) of the participants were exposed to SHS. Exposure to SHS was associated with an estimated 2.9 g/m^2^ increase in LV mass index, with a dose-response relationship between higher serum cotinine and LV mass index. These results were consistent in men and women, Whites and non-Whites, elderly and non-elderly, and those with and without hypertension. Significant effect modification was present among obese individuals with an estimated 4.8 g/m^2^ increase in LV mass index (interaction p=0.01).

**CONCLUSIONS:**

In a racially diverse sample of non-smokers, SHS is associated with increased LV mass with a dose-response relationship between level of exposure and LV mass. Effect modification was present among obese individuals. These findings underscore the harmful effect of passive smoking on the cardiovascular system and highlight the need for more restrictions on smoking in public areas, especially in countries or regions with less-stringent public health policies.

## INTRODUCTION

Cigarette smoking is a well-described health hazard. Less is known about the harms of secondhand smoke (SHS) exposure, but a growing body of evidence has identified it as a major public health problem and it is estimated to cause 1% of total worldwide mortality^1^. Recent evidence suggests that SHS exposure is associated with chronic hypertension among non-smokers^2^. Hypertension affects about one-third of US adults and is a well-recognized risk factor for cardiovascular disease (CVD), end-stage renal disease, and increased mortality^3-5^. Globally, tobacco use and hypertension are the two leading risk factors for preventable mortality and attributable disability-adjusted life years, so SHS exposure has the potential for enormous public health consequences^2,6^.

Since hypertension is the strongest risk factor for the development of left ventricular hypertrophy (LVH)^7,8^, it would follow that SHS exposure would also be a risk factor for LVH. This has been examined in a single animal study where SHS exposure induced a pro-inflammatory response resulting in concentric transition from eccentric LVH in old hamsters^9^. However, to date, there have not been any clinical studies measuring this association.

Cotinine, a metabolite of nicotine, is a highly sensitive and specific biomarker for recent tobacco smoke exposure and is detectable in non-smokers who have been exposed to SHS^10,11^. As such, cotinine can be used as an objective surrogate for SHS exposure^11^. Since SHS exposure is associated with chronic hypertension, we hypothesized that SHS exposure would also be associated with increased left ventricular (LV) mass. We tested this hypothesis in a cross-sectional cohort study to assess the relationship of SHS exposure as measured by serum cotinine and electrocardiographic (ECG)-assessed LV mass index in non-smoker participants.

## METHODS

The Third National Health and Nutrition Examination Survey (NHANES-III) is a survey of a representative sample of the civilian US population that provides estimates of disease prevalence and overall health status^12^. All participants provided written consent at the time of study enrollment. Data in NHANES-III were collected from 1988–1994 through an in-home interview process as well as a subsequent appointment at a mobile examination center. Venipuncture was also performed at the time of interview by a trained technician for blood sample analysis.

We only included patients with ECG data in our analysis. We excluded patients who were self-reported current smokers. Active smokers almost always have serum cotinine ≥10 ng/mL, so only participants with serum cotinine ≤10 ng/mL were included^13,14^. Patients with poor quality ECGs or major intraventricular conduction delay were excluded. Participants with atrial fibrillation were not excluded. Of the 8561 participants with ECG data, 4982 were included in the final analysis after exclusions.

Age, gender, race (White, Black, and other), smoking status, and history of CVD (heart failure, stroke, or heart attack) were defined by self-report. Diabetes was defined as fasting serum glucose ≥126 mg/dL, hemoglobin A1c ≥6.5%, or use of an antihyperglycemic medication^15^. Hypertension was defined as systolic blood pressure ≥130 mmHg or diastolic blood pressure ≥80 mmHg according to American Heart Association/American College of Cardiology guidelines^16^ or use of an antihypertensive medication. Hyperlipidemia was defined as total cholesterol ≥200 mg/dL, serum triglycerides ≥150 mg/dL, or use of lipid-lowering medications.

Serum cotinine was measured by isotope-dilution-high-performance liquid chromatography atmospheric pressure chemical ionization-tandem mass spectrometry^17^.

Twelve-lead ECGs were obtained by trained technicians with a Marquette MAC 12 system (Marquette Medical Systems, Milwaukee, WI) during mobile examination visits. Analysis of ECGs was achieved through an automated computerized process and visual inspection by a trained technician in a centralized core laboratory. ECG-LV mass index (ECG-LVMI) was estimated using an electrocardiographic model developed and applied in NHANES-III^18^ then validated in the Cardiovascular Health Study^19^.

Baseline characteristics were compared between those with SHS exposure and those without. SHS exposure was defined as serum cotinine ≥1 ng/ mL which is considered the cut-off for ‘heavy’ SHS exposure by the Centers for Disease Control^14^. Continuous variables were reported as mean ± standard deviation. Categorical variables were reported as frequency and percentage. Student’s t-test was used to compare continuous variables and a chi-squared test was used to compare categorical variables.

Multivariable linear regression was used to estimate the association between ECG-LVMI and SHS using non-standardized beta coefficients. The association of ECG-LVMI and serum cotinine was also measured across serum cotinine quartiles. In both methods, Model 1 was adjusted for demographics such as age, gender, and race; and Model 2 was adjusted as for Model 1 plus known CVD risk factors including hypertension, diabetes, hyperlipidemia, and history of CVD. Obesity was not included in the model since ECG-LVMI is already adjusted for body mass.

To examine consistency across subgroups, the association between ECG-LVMI and serum cotinine quartiles was evaluated when stratified by age (<60 vs ≥60 years), race (White vs non-White), gender (male vs female), hypertensive status (hypertensive vs non-hypertensive), and obesity (obese vs non-obese). Interaction was tested for using variables similar to those used in Model 2 with addition of the interaction term between serum cotinine quartile and subgroup stratification.

All statistical analyses were conducted using R version 4.0.3^20^ and p was considered significant if <0.05.

## RESULTS

There were 4982 non-smoker participants included in the analysis (60.8 ± 13.6 years, 57.8% women, 77.7% Whites). Table 1 shows the characteristics of the participants stratified by SHS exposure status. Participants with SHS exposure were more likely aged <60 years, male, non-White, and diabetic. They also had higher diastolic blood pressure.

**Table 1:** Baseline characteristics

*Characteristics*	*Secondhand smoke exposure*		*p*
	*Present (n=489)*	*Absent (n=4493)*	
	*Mean ± SD or n (%)*	*Mean ± SD or n (%)*	
**Serum cotinine** (ng/mL)	2.33 ± 1.59	0.20 ± 0.21	<0.001
**Age** (years) ≥ 60	222 (45.4)	2452 (54.6)	<0.001
**Men**	236 (48.2)	1864 (41.5)	0.004
**Race**			<0.001
White	306 (62.6)	3567 (79.4)	
Black	163 (33.3)	795 (17.7)	
Other	20 (4.1)	131 (2.9)	
**Hypertension**	330 (67.5)	2950 (65.7)	0.45
**Obesity**	168 (34.3)	1362 (30.3)	0.07
**Systolic blood pressure** (mmHg)	134.0 ± 23.4	134.0 ± 22.8	0.98
**Diastolic blood**	75.9 ± 12.7	73.9 ± 13.2	0.001
**pressure** (mmHg)			
**Antihypertensive medication**	116 (23.7)	1110 (24.7)	0.67
**Diabetes**	103 (21.1)	747 (16.6)	0.016
**Hyperlipidemia**	370 (75.7)	3389 (75.4)	0.95
**Cardiovascular disease**	55 (11.2)	499 (11.1)	0.98

Diabetes was defined as fasting serum glucose ≥126 mg/dL, hemoglobin A1c ≥6.5%, or use of an antihyperglycemic medication. Hypertension was defined as systolic blood pressure ≥130 mmHg or diastolic blood pressure ≥80 mmHg or use of an antihypertensive medication. Obesity was defined as body mass index >30 kg/m^2^. Hyperlipidemia was defined as total cholesterol ≥200 mg/dL, serum triglycerides ≥150 mg/dL, or use of lipid-lowering medications.

In a linear regression model adjusted for demographic and co-morbid covariates, SHS was significantly associated with a 2.9 g/m^2^ increase in ECG-LVMI. When measured across serum cotinine quartiles, the highest quartile of serum cotinine was associated with a 2.3 g/m^2^ increase in ECG-LVMI when compared to the lowest quartile (Table 2).

**Table 2:** Linear regression between SHS exposure and serum cotinine quartiles with LV mass index

	*Level/group*	*Serum Cotinine (ng/mL)*	*Participants (n)*	*Model 1 Age + sex + race*		*Model 2 Model 1 + hypertension + diabetes + hyperlipidemia + prior CVD*	
				*LV mass index (g/m^2^) Beta coeff (95% CI)*	*p*	*LV mass index (g/m^2^) Beta coeff (95% CI)*	*p*
**SHS exposure**	Absent (Ref.)	<1.0	4493	Ref.	-	Ref.	-
	Present	1.0–9.91	489	3.38 (1.45–5.31)	<0.01	2.93 (1.03–4.83)	<0.01
**Cotinine quartiles**	Q1 (Ref.)	<0.06	1238	Ref.	-	Ref.	-
	Q2	0.06–0.14	1254	0.09 (-1.53–1.71)	0.91	0.40 (-1.19–2.00)	0.62
	Q3	0.14–0.36	1247	0.64 (-1.00–2.27)	0.45	0.80 (-0.81–2.42)	0.33
	Q4	0.36–9.91	1243	2.55 (0.88–4.22)	<0.01	2.30 (0.66–3.94)	<0.01

Beta coeff: non-standardized beta coefficient. CVD: cardiovascular disease. LV mass: left ventricular mass. SHS: secondhand smoke. Ref.: reference.

Table 3 shows the association between SHS and ECG-LVMI across subgroup stratifications. Stratification by age (interaction p=0.94), race (interaction p=0.75), gender (interaction p=0.40), and hypertensive status (interaction p=0.19) showed consistent results among groups analyzed. Significant effect modification was present when stratified by obesity (interaction p=0.01) and showed a 4.8 g/m^2^ increase in ECG-LVMI.

**Table 3:** Linear regression between SHS exposure with LV mass index in subgroups

*Subgroup*		*Model 1 Age + sex + race*	*Model 2 Model 1 + hypertension + diabetes + hyperlipidemia + prior CVD*	
		*LV mass index (g/m^2^) Beta coeff (95% CI)*	*LV mass index (g/m^2^) Beta coeff (95% CI)*	Interaction p*
**Age** (years)	≥60	3.82 (0.70–6.94)	3.33 (0.26–6.41)	0.94
	<60	2.93 (0.67–5.19)	2.55 (0.33–4.77)	
**Race**	Non-White	-0.28 (-4.06–3.51)	-0.82 (-4.52–2.89)	0.40
	White	3.97 (1.68–6.27)	3.59 (1.34–5.85)	
**Sex**	Men	1.77 (-1.03–4.57)	1.32 (-1.43–4.08)	0.75
	Women	3.71 (1.13–6.30)	3.26 (0.72–5.79)	
**Hypertension**	Present	3.99 (1.52–6.45)	3.80 (1.35–6.26)	0.19
	Absent	1.35 (-1.56–4.27)	1.04 (-1.85–3.93)	
**Obesity**	Present	4.93 (2.56–7.30)	4.79 (2.46–7.13)	0.01
	Absent	0.31 (-3.00–3.62)	-0.62 (-3.87–2.63)	

Beta coeff: non-standardized beta coefficient. CVD: cardiovascular disease. LV mass: left ventricular mass. SHS: secondhand smoke.

* Interaction p calculated from Model 2.

## DISCUSSION

In this racially diverse analysis of non-smoker participants, we observed a positive association between cotinine-verified SHS exposure and ECG-LVMI; this relationship appeared to be consistent across subgroups stratified by age, race, gender, and hypertensive status. Significant effect modification was present when stratified by obesity. Since obese individuals are known to have elevated baseline risk for increased LV mass^21^, our findings suggest that this is a high-risk group that is more susceptible to the effects of SHS exposure.

When examining SHS exposure by self-report, the degree of exposure is subject to multiple forms of reporting bias. A single study of the US workforce found that self-reported denial of SHS exposure was only about 28% accurate^22^. Another study also demonstrated that self-reported SHS exposure significantly underestimated actual SHS exposure as determined by cotinine verification^23^. A significant advantage of using serum cotinine is that it allows for an objective measure of the degree of exposure at the time of measurement. It has been effectively used as a biomarker for exposure to tobacco in both active and SHS exposure due to its long half-life^10,11^. It is generally accepted that active smokers nearly always have serum cotinine ≥10 ng/mL and is often higher than 500 ng/mL^14^. Typical exposure is generally ≤1 ng/mL and heavy exposure is in the 1–10 ng/mL range^13,14^. Thus, in this study, we examined cotinine levels both in quartiles and relative to 1 ng/mL among non-smokers and excluded those with serum cotinine ≥10 ng/mL. We observed a significant association between ECG-LVMI and SHS exposure after adjusting for other known risk factors for increased LV mass including hypertension, diabetes, gender, race, hyperlipidemia, and history of CVD^24-27^.

Even though LVH had been observed in hamsters with SHS exposure, to our knowledge, our report is the first to examine this association in a clinical study. Our study has important clinical implications since increased LV mass has well-known prognostic significance. In observational studies, increased LV mass is the strongest independent predictor of CVD events aside from age, with an average risk ratio of 2.5 for overall CV events including myocardial infarction, heart failure, stroke, and death^28,29^. The most concerning of these associations is the connection between increased LV mass and arrhythmogenesis; it is associated with atrial fibrillation, ventricular tachycardia, ventricular fibrillation, and sudden cardiac death^7^. The mechanism behind this is likely the result of diverse etiologies and is characterized by pathological changes in myocardial tissue on the genetic, molecular, cellular, and tissue level. Currently, it is thought that this process is mediated by myocardial cell hypertrophy without corresponding increases in cell numbers, leading to increased fibroblasts, interstitial collagen accumulation, fibrosis, maladaptive remodeling, and finally myocardial structural disarray and consequent conductive aberrancy^7,30^. This is further influenced by neurohormones and cytokines^31^.

The specific mechanism behind this association is unknown; *in vitro* data suggest that the chemical composition of cigarette smoke (e.g. carbon monoxide, nitrosamines, polycyclic aromatic hydrocarbons) induces a pro-inflammatory state evidenced by increases in TNF-α and IL-1β^32,33^. In theory, the pro-inflammatory effect of cigarette smoke on myocardial tissue may promote the cellular hypertrophy, fibrosis, and structural abnormalities that ultimately lead to increased LV mass and its downstream sequelae^9^. SHS exposure is also associated with increased oxidative stress, endothelial dysfunction, arterial stiffness, and imbalance between sympathetic and parasympathetic control; all can contribute to LVH^2,34,35^.

Given that our study suggests that SHS exposure is associated with increased LV mass independent of other known risk factors, this is further substantiation behind the need for more restrictions on smoking in public areas. This is especially important in low- and middle-income countries and poor communities since these regions have been shown to have the highest levels of SHS exposure^36,37^, so policies targeting these areas would likely experience the largest health benefits.

### Strengths and limitations

Our study has several limitations. The cross-sectional design of the analysis is subject to temporality and residual confounding. In addition, the measurement of serum cotinine was obtained during the time of laboratory collection only, so this may not represent the true degree of exposure in a given participant. ECG was used to estimate the LVMI, however, the gold standard of LV mass measurement is cardiac MRI; ECG-LVMI is significantly less sensitive^38^. Additionally, ECG-LVMI measurements were validated based on echocardiography, which may overestimate the degree of LV mass^19^. Further, participants with high levels of physical activity have been shown to have increased LV mass^39^, so this is another potential confounder we were unable to adjust for.

Strengths of our study include a large sample size and racially diverse population. Our measure of SHS exposure is also a strength since we were able to provide an objective level of exposure based on serum cotinine levels. In addition, since it is unlikely for SHS exposure to cause serum cotinine ≥10ng/mL in true non-smokers, we excluded participants who may have had questionable self-reported smoking status^14^. Despite the limitations of this study, our results indicate a novel association that can be considered a first approach of a more complete investigation using a prospective cohort or other study design.

## CONCLUSIONS

In a racially diverse sample of non-smokers, SHS is associated with increased LV mass with a dose-response relationship between level of exposure and LV mass. There was also significant effect modification present among obese participants. These findings underscore the harmful effect of passive smoking on the cardiovascular system and highlight the need for more restrictions on smoking in public areas, especially in countries or regions with less-stringent public health policies. Further, the effect modification by obesity merits a personalized risk assessment when counseling patients on potential harms of SHS exposure.
